# A Secure Video Steganography Based on the Intra-Prediction Mode (IPM) for H264

**DOI:** 10.3390/s20185242

**Published:** 2020-09-14

**Authors:** Mingyuan Cao, Lihua Tian, Chen Li

**Affiliations:** School of Software Engineering, Xi’an Jiaotong University, Xi’an 710049, China; laocao81@stu.xjtu.edu.cn (M.C.); cclidd@xjtu.edu.cn (C.L.)

**Keywords:** video steganography, intra-prediction mode, H264/AVC, security

## Abstract

Recently, many video steganography algorithms based on the intra-prediction mode (IPM) have been adaptive steganography algorithms. These algorithms usually focus on the research about mapping rules and distortion functions while ignoring the fact that adaptive steganography may not be suitable for video steganography based on the intra-prediction mode; this is because the adaptive steganography algorithm must first calculate the loss of all cover before the first secret message is embedded. However, the modification of an IPM may change the pixel values of the current block and adjacent blocks, which will lead to the change of the loss of the following blocks. In order to avoid this problem, a new secure video steganography based on a novel embedding strategy is proposed in this paper. Video steganography is combined with video encoding. Firstly, the frame is encoded by an original encoder and all the relevant information is saved. The candidate block is found according to the relevant information and mapping rules. Then every qualified block is analyzed, and a one-bit message is embedded during intra-prediction encoding. At last, if the IPM of this block is changed, the values of the residual are modified in order to keep the optimality of the modified IPM. Experimental results indicate that our algorithm has good security performance and little impact on video quality.

## 1. Introduction

Information hiding is the method to keep information secure, and because of its great performance, information hiding has become more and more popular all over the world [1,2,3]. As one of the information hiding techniques, steganography embeds the secret message into the cover, affecting the cover only a little. In addition, only the recipient is allowed to detect the embedding event. So, the most important performance of an excellent steganography is security. In the meantime, steganalysis is designed for detecting the presence of a hidden message [4,5,6]. 

Steganography is widely used in digital media, such as image, audio and video [7,8,9]. In the past, many scholars focus on image steganography while only a few scholars are interested in video steganography. With the development of multimedia and the internet, video applications become more and more popular all over the world. In addition, video steganography has a large capacity and less distortion. Hence, video steganography has drawn more and more interest over the last few years. Generally speaking, there are four kinds of video steganography based on different positions: the intra-prediction mode [10,11], motion vector [12,13], Discrete Cosine Transform (DCT) coefficients [14,15] and entropy coding coefficient [16]. Because video steganography based on DCT coefficients and entropy coefficients considerably affect the video quality, only a few scholars focus on them. Most of the scholars focus on video steganography based on IPM and motion vector. Compared with inter-encoding, though, intra-encoding has a low computational complexity and low efficiency, which means intra-encoding will cost more bits to code one frame. On the other hand, it means the intra-frame has more redundant information when hiding secret messages.

In the beginning, adaptive steganography was proposed for image information hiding; many years later, because of its great performance, adaptive steganography has become the most important image steganography technology [17,18]. More than that, nowadays, adaptive steganography technology is widely using in video steganography, too. There are many adaptive steganography algorithms based on both intra-prediction mode and motion vector. Some of them have great security and affect the video quality only a little, so adaptive steganography technology has become more and more popular in information hiding area, including video steganography based on IPM.

The first video steganography algorithm was proposed by Hu et al. [19]. The secret message is embedded into the qualified IPM based on the mapping rules; but, the number of bits may increase along with the growth of the embedding strength because of the algorithm structure. After that, Yang et al. [20] improved the steganography algorithm proposed by Hu. In this paper, a video steganography based on the intra-prediction mode and matrix coding is proposed, and all of the qualified IPMs are divided into groups: two-bit secret messages are embedded into every three IPMs by matrix coding. The security of this algorithm is better because of the embedding position template. Bouchama et al. [21] proposed a new data-hiding approach by exploiting the IPMs for 4 × 4 luminance blocks. Firstly, the IPMs are divided into four groups, and then in order to maintain video quality, a secret message is embedded by modifying the modes of the same group. Although this algorithm has huge capability, it cannot resist the detection of the steganalysis algorithm. Zhang et al. [22] presented an adaptive video steganography based on IPM for H264/AVC. In this paper, the proposed cost assignment scheme is based on the statistical minimum residual between the sub-optimal Sum of Absolute Transformed Difference (SATD) and optimal SATD. Then, the qualified IPMs are chosen to be modified by the Syndrome-Trellis Code (STC). Experiments indicate that this algorithm has good performance against the steganalysis algorithm. Nie et al. [23] proposed an effective adaptive video steganography based on the intra-prediction mode. In this paper, the sum of the absolute different prediction deviation is used to define the distortion function. Then, the mapping rule for each frame is generated statistically by SAD prediction deviation (SPD) information. At last, Syndrome-Trellis Codes are used to choose which IPM need to be modified. Experimental results indicate that this algorithm has great performance in resisting steganalysis. 

The review above shows that the existing video steganography based on IPM is mainly the adaptive steganography algorithm. Most of them focus on the mapping rule and distortion function for the Syndrome-Trellis Code while ignoring the fact that adaptive steganography may not be suitable for video steganography based on the intra-prediction mode. In order to avoid this problem, a secure video steganography based on the intra-prediction mode is proposed in this paper. Firstly, the frame is encoded by the original encoder and all the relevant information is saved. Then, every qualified block is analyzed, and embedded in a one-bit message during intra-encoding. At last, if the IPM of this block is changed, all the values of the residual are modified to zero in order to keep the optimality of the modified IPM. In this way, we can ensure the modified IPM will keep its optimality after embedding. Hence, this algorithm can effectively resist the steganalysis algorithm. 

In summary, this paper makes three novel contributions:

1. The modification of an IPM may change the pixel values of the current block and adjacent blocks, which will lead to a change in the cost of the following blocks; in order to avoid this problem, a new secure video steganography based on a novel embedding strategy is proposed in this paper.
2. Because not all the blocks are appropriated for embedding messages, cover selection rules are proposed in this paper; all the blocks are analyzed and only the qualified block is embedded during the intra-prediction encoding.
3. In order to avoid detection by steganalysis, after modifying the IPM of the selected block, all the residual values of the same block are modified to maintain the optimality of the modified IPM.

The rest of this paper is organized as follows. The process of intra-prediction is briefly introduced in Section 2. Then, the details of the proposed video steganography are presented in Section 3. In Section 4, a summary of the embedding and extraction procedure is presented. In Section 5, the experimental evaluation and analysis are presented. Finally, Section 6 gives the conclusion of this paper.

## 2. Intra-Prediction Coding Scheme in H264 

As an open video coding standard, H264/AVC was developed by the ISO/IEC MPEG and the ITUT VCEG standards committees [24]. High data compression efficiency is the biggest advantage of AVC. In addition, H.264 provides smooth, continuous and high-quality frames. Besides the hybrid coding structure of the DPCM differential coding and DCT transform coding, AVC adds new coding methods, such as intra-prediction, multi-frame prediction, multi-mode motion estimation, 4 × 4 integer transform and content-based variable length coding to improve the coding efficiency. The video steganography is processed during the encoding of H.264, and the covers of the video steganography are based on the major features of the H.264/AVC standard; hence, there are four kinds of video steganography: the intra-prediction mode, motion vector, DCT coefficients and entropy coding coefficient.

In order to transmit and store easily, uncompressed video can effectively compress into a smaller video file by using video-coding techniques. For the uncompressed video sequence, there is temporal redundancy between the adjacent frames and special redundancy in a single frame. Spatial redundancy indicates that the values of the adjacent pixels are similar in the same frame, in other words, the adjacent regions usually have a high correlation. Therefore, decreasing the spatial redundancy is an effective method to obtain a high compression rate in the intra-prediction technique. There are four kinds of IPM in AVC, intra 4 × 4, intra 16 × 16, intra chroma and I_PCM. The intra 4 × 4 blocks are widely used in video steganography based on intra-prediction modes. Because the detail areas are usually encoded in intra 4 × 4 blocks, embedding messages in intra 4 × 4 blocks will have less influence on the video quality.

The basic procedure of the intra-prediction scheme in AVC is proposed as follows:

Firstly, the most probable mode (MPM) is calculated based on the IPMs of the adjacent blocks in Formula (1). $M_{up}$ and $M_{left}$ are the optimal IPMs of the adjacent blocks, which are already encoded.

(1) $$\mathrm{MPM}=min\left\{M_{up},M_{left}\right\}$$

Then, all the rate distortion (RD) costs of the candidate IPMs are calculated by using Formula (2). Parameter λ represents the Lagrange multiplier for intra-prediction. *R* is the number of bits to encode the block. Since the computation complexity of RDcost is high, SATD is another choice to select the optimal IPM. The formula of SATD is shown in Formula (3). *H* represents Hadamard Transform. The intra-prediction mode with minimum cost will be chosen as the optimal IPM of the current block.

(2) $$\mathrm{RDcost}=\underset{1\leq x,y\leq 4}{\sum }\left|S_{i}^{REC}\left(x,y\right)-S_{i}^{ORI}\left(x,y\right)\right|+\lambda R$$

(3) $$\mathrm{SATD}=\underset{1\leq x,y\leq 4}{\sum }\left|H(S_{i}^{REC}\left(x,y\right)-S_{i}^{ORI}\left(x,y\right))\right|$$

$S_{i}^{REC}\left(x,y\right)$ is the pixel values of the reconstructed blocks on coordinate (*x*, *y*).$\;S_{i}^{ORI}\left(x,y\right)$ denotes the pixel values of the original blocks on coordinate (*x*, *y*).

Finally, the identifier pre is calculated based on the optimal IPM and MPM of the current block. If the IPM and the MPM of the current block are the same, the pre will be set to 1, and then the optimal IPM will not be stored in order to save the bits of the coding stream. If the IPM and the MPM of the current block are different, the pre will be set to 0, and then optimal IPM will be stored in the coding stream. So, it will have great influence on the number of bits, if the IPM is changed in the block with the pre equaling 1.

## 3. The Proposed Algorithm

In this section, firstly, we analyze the influence of different embedding orders to the performance of steganography based on the intra-prediction mode. Then, the influence of optimality on the performance of the steganography based on the intra-prediction mode is analyzed. 

### 3.1. The Influence of a Different Embedding Strategy

Recently, adaptive steganography has been widely used in video steganography based on the intra-prediction mode because of its great performance. There are many adaptive steganography algorithms based on both the intra-prediction mode and motion vector. Some of them have great security and little influence on the video quality, so adaptive steganography technology become more and more popular in information hiding area. Although adaptive steganography technology may suit for image steganography and video steganography based on motion vector, but adaptive steganography technology is not the perfect match for video steganography based on the intra-prediction mode.

Because that adaptive steganography must calculate the cost of modifying every cover in order to choose the appropriate covers to modify. In all of the adaptive steganography algorithms, the definition of the distortion function is the most important part. Each author tries to define the distortion function in different ways. However, those facts exist with one condition: the changing of one cover has little influence on the rest of the covers. Otherwise, the loss of the rest of the covers may change after the first cover is modified. In addition, this distortion may accumulate after more covers are modified. There are some adaptive steganography algorithms based on the intra-prediction mode that exist, all of them must calculate the cost of modifying every cover before embedding the first secret message to the cover. However, in AVC intra-prediction, every block is encoded based on several pixel values of its adjacent blocks, which means the best intra-prediction mode of the same block may change if some pixel values of its adjacent blocks have a little variation. If some bits of a secret message are embedded into the cover blocks, the distortion of the rest of the cover blocks may have a few differences from the distortion they calculate before. In addition, this distortion may accumulate after more covers are modified just like the intra-frame distortion drift. In summary, adaptive steganography technology is not the perfect choice for video steganography based on the intra-prediction mode because of its embedding strategy. 

**Figure 1:**
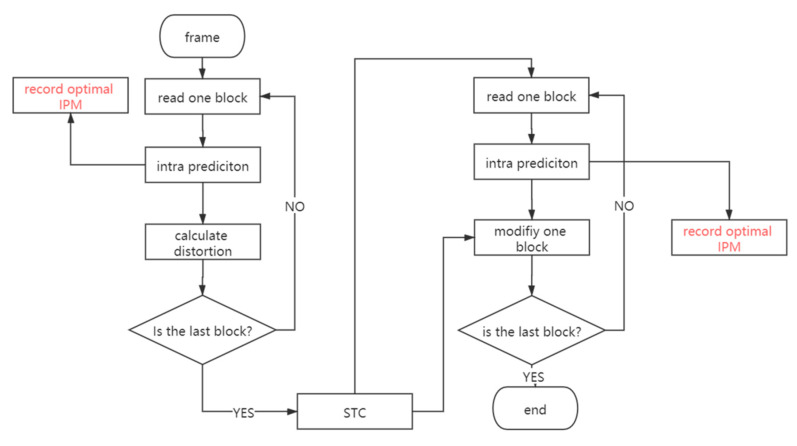
Experiment about the adaptive steganography embedding strategy.

As a typical adaptive video steganography based on the intra-prediction mode, this paper selects Nie et al.’s algorithm to verify the above viewpoint. As shown in Figure 1, the procedure of embedding is not changed while the optimal IPMs of every cover are recorded before the calculation of the embedding distortion definition. After executing the STC, the optimal IPMs of the same blocks are also recorded before the modification is done. 

Ten video sequences were tested in different quantization parameters (QPs). The ratio of the optimal IPM changing is shown in Table 1. The experimental result shows that almost 20% of the optimal IPMs are already changed before the modification. It means at least 10% loss of the covers are changed before the modification; thus, adaptive steganography is not the best choice for video steganography based on the intra-prediction mode. In order to solve this problem, a different embedding strategy is proposed in this paper. Different from adaptive steganography, the modification of every cover is executed right after the selection of every cover. So, the modification will not affect the embedding of the rest of the covers. It makes the steganography more effective against steganalysis. In summary, a secure steganography algorithm based on IPM must modify the IPM of the current block immediately before the intra-prediction of the next block.

### 3.2. The Influence of Optimality of the IPM

In video steganography based on IPM, the IPMs of the selected blocks are modified in order to embed a secret message, which means the optimal IPM of the cover block is modified to a suboptimal IPM. So, the optimality of these IPMs will be destroyed. This is also the main idea for steganalysis to decide whether the video is embedded with a secret message or not. In order to avoid detection by steganalysis, the IPM of the cover blocks should try to maintain its optimality while embedding a secret message. 

To achieve this purpose, after modifying the IPM of a selected block, all the residual values of the same block are modified to zero. Since all the residual values are modified to 0, the values of the reconstruction pixels and the values of the prediction pixels with the current IPM are the same. In other words, the current IPM will be the optimal IPM for the cover block in view of the steganalysis algorithm. So, the information-hiding algorithm has great performance against the steganalysis.

## 4. Procedure of Embedding and Extraction

In order to make our algorithm more secure, an appropriate embedding strategy was chosen. Because the pixel values of the current block are predicted by pixel values of the adjacent blocks in the intra-prediction encoding scheme, the modification of the IPM must processed immediately before the intra-prediction of the next block. Otherwise, the statistical information may change after the modification of the IPMs. In addition, keeping the optimality of the IPMs can improve the security of the steganography algorithm. In this paper, the residual values of the current block will be modified after the message is embedded in the current block. Besides, the mapping rule and cover selection is important to the performance of our algorithm; the procedure of the mapping rule and cover selection is proposed in Section 4.1 and Section 4.2. The procedure for message embedding and extraction is presented in Section 4.3 and Section 4.4.

### 4.1. The Mapping Rule

In order to embed the secret message to the IPM of every selected cover, all nine IPMs are divided into two groups. These two groups represent two different kinds of secret messages, 0 and 1. If the secret message is 0, the IPM of this cover should modify to the IPM in Group 0. If the secret message is 1, the IPM of this cover should modify to the IPM in Group 1. In AVC intra-encoding, there are nine IPMs that exist, and a similar direction means a similar prediction method. Because the cover IPM will not be modified to the IPM in the same group, the similar prediction method should not exist in the same group. Based on this, all nine IPMs are divided into these two groups in Formula (4). If the secret message of 0 is embedded into the blocks, the IPM of this block will modify to IPM that has the minimum cost in Group 0.

(4) $$\{\begin{array}{c} Group\;0:Mode\;\;0\;1\;3\;4\;\\ Group\;1:Mode\;2\;5\;6\;7\;8\;\;\;\; \end{array}$$

### 4.2. Cover Selcection Rule

As we all know, there are two different blocks that exist in AVC intra-macroblock. In this paper, only intra 4 × 4 blocks are chosen to embed the messages while the IPMs of the intra 16 × 16 blocks are not modified; this is because there are nine available IPMs in the intra 4 × 4 block while only four available IPMs in the intra 16 × 16 block. The more available the IPMs, the more difficult for the steganalysis to detect the video cover accurately. Furthermore, a smooth area is usually encoded in the intra 16 × 16 blocks while the detail area is encoded in intra 4 × 4 blocks. So, embedding messages in intra 4 × 4 blocks will have less influence on the video quality. The secret messages are only embedded into the intra 4 × 4 blocks.

Not all the intra 4 × 4 blocks are appropriated for embedding messages in this paper. There are some blocks whose residual values are almost zero. These blocks are appropriated to embed the secret messages naturally; but, there are some blocks whose residual values are quite complicated and large. Embedding message in these blocks will have great influence on the video quality. In order to keep a balance between security and video quality, there is another cover selection rule. Firstly, assuming the coding of the intra 4 × 4 blocks are going to embed a one-bit message, the modified IPM can be decided based on the cost order and mapping rule. Then, the sum of all absolute values of the inverse transform residual matrix with the modified IPM are calculated before the modification, given as S. If S is greater than the threshold T, this block will embed a one-bit message, and all the residual values are modified to 0. If S is smaller than the threshold T, this block will not embed any message. Hence, the messages will embed into the blocks with little influence on the video quality. The modified IPM will still be the optimal IPM after encoding since the residual values are modified to zero.

### 4.3. Procedure of Embedding

**Figure 2:**
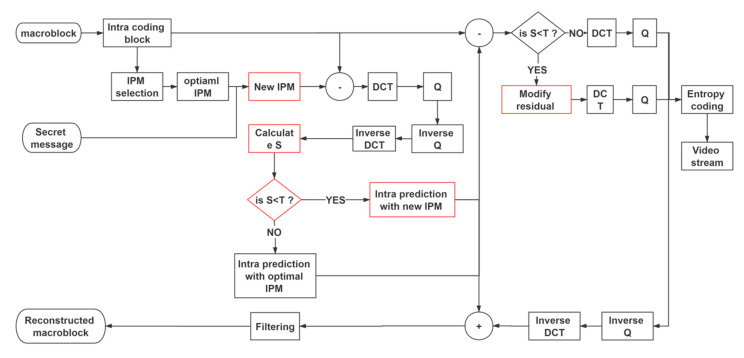
Procedure of embedding a message.

According to Section 3 and Section 4, the steps of message embedding are illustrated in Figure 2.

1. One frame of the original video is read.
2. Read one intra 4 × 4 block from the encoding frame.
3. Firstly, assuming the coded intra 4 × 4 blocks are going to be embedded as one-bit messages, the modified IPM can be decided based on the cost information and mapping rule.
4. Then, the sum of all absolute values of the inverse DCT transform residual matrix with the modified IPM is calculated before the modification.
5. Decide whether this block is going to be embedded as a one-bit secret message or not. The embedding position is recorded and encrypted by a private key. If the block can meet the cover selection rule, then the IPM will be modified and all the residual values are modified.
6. Repeat Step 2 until all the intra 4 × 4 blocks are processed.

### 4.4. Procedure of Extraction

The process of message extraction is presented as follow:

1. One frame of the original video is read.
2. Read one intra 4 × 4 block from the encoding frame.
3. The position of the embedding blocks is generated by private key, the message is extracted by mapping rule.
4. Repeat Step 2 until all the secret messages are extracted.

## 5. Experimental Results

In this section, in order to measure the performance of the proposed video steganography algorithm, experimental results are presented as follow. A video database [25,26,27,28] containing 17 YUV video sequences in CIF format was used for the experiments. These 17 video sequences were divided into 178 subsequences without overlapping while each subsequence contains 60 frames. The maximum interval of the intra-frame was set to 12. The embedding scheme was implemented by JM, and the LibSVM toolbox was used for the classification. In order to evaluate the performance of the proposed algorithm, Nie et al.’s algorithm, Bouchama et al.’s algorithm and Wang et al.’s algorithm [29] were chosen to be compared with ours.

### 5.1. Threshold

In our algorithm, the threshold T was used in cover selection, which has a great influence on the performance of the algorithm. When T becomes larger, more blocks will participate in the information embedding, thus the embedding capability of our algorithm will increase. However, since the values of the residual matrix will be modified after the message is embedded in the current block, the video quality will decrease if T becomes larger. In order to test the influence of different thresholds, experiments are carried out for different thresholds. Table 2, Table 3 and Table 4 show the security of the steganography algorithm under different thresholds. As shown in Table 2, on the surface, the security decreases with the increase of the threshold. However, the security of the steganography should be compared with a similar capability. When T increases, the embedded capacity also increases. Therefore, considering both accuracy and embedding capability, our algorithm can maintain the security under different thresholds. At the same time, we also give the effect on video quality under different thresholds.

In this experiment, video quality is evaluated by power signal-to-noise ratio (PSNR). PSNR is measured by Formulas (5) and (6). As shown in Table 3, it seems that the change in threshold has little effect on video quality. However, the video quality should decrease while the threshold is increasing theoretically. That is because when we calculate PSNR in MSU Video Quality Measurement Tool, the result is the average PSNR of all the video frames, including both P frames and I frames. But in video steganography based on IPM, information is only embedded in I frames. Table 4 shows the PSNR of the instantaneous decoding refresh (IDR) frame under different thresholds. As can be seen from Table 4, the threshold has a great influence on the IDR frame. This is consistent with the theory. Considering all the experimental results, the video quality of the I frame basically depend on the threshold, while the security can maintain great performance with different thresholds. In order to keep the balance between the video quality and security, the threshold T is set to 48 in the following experiments.

(5) $$\mathrm{PSNR}=10\times \log_{10}\left(\frac{\mathrm{MAX}}{\mathrm{MSE}}\right)$$

(6) $$\mathrm{MSE}=\frac{1}{\mathrm{mn}}\overset{m-1}{\underset{i=0}{\sum }}\overset{n-1}{\underset{j=0}{\sum }}{\left[I\left(i,j\right)-K\left(i,j\right)\right]}^{2}$$

### 5.2. Video Quality

In order to evaluate the performance of the steganography algorithm, PSNR is used to measure the video quality with a different bit rate. PSNRs are widely used to evaluate video quality in the steganography algorithms.

**Figure 3:**
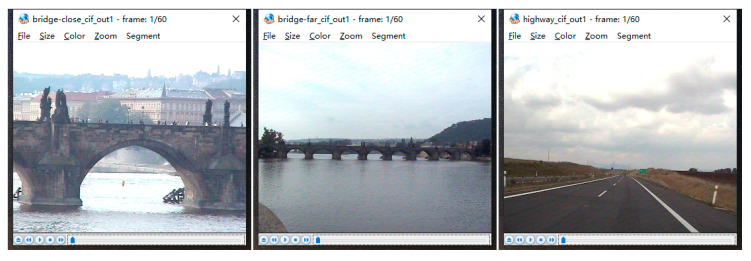
Original video frame.

**Figure 4:**
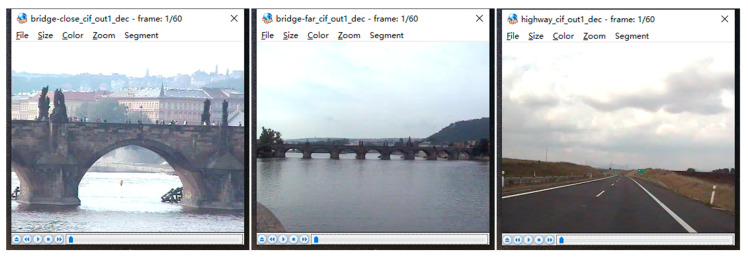
Video frame after embedding.

Different encoding standards have great influence on the video quality; so, Wang et al.’s algorithm does not participate in this experiment because this algorithm is based on the HEVC encoding standard while the other algorithms are based on the AVC encoding standard. In this experiment, several video sequences were compressed by different steganography algorithms. The result is shown in Table 5. Since our algorithm will change both the IPM and residual matrix during the message embedding, there will be much distortion of the video streams. Though the results show that our algorithm has the least performance in video quality, the difference between the other algorithms and our algorithm is not huge. As shown in Figure 3 and Figure 4, it is not easy for everyone to tell the difference between the original video and the video after embedding.

### 5.3. Security

Security is the most important issue for all video steganography algorithms. In order to measure the security of the steganography, a state-of-the-art steganalysis was processed. Because of its great detection performance against video steganography based on IPM, Zhao et al.’s algorithm [30] was chosen to detect all the video sequences. The security performance was evaluated by the accuracy rate (AR), which can be calculated by Formula (7):

(7) AR=(TP+TN)÷2

The true positive rate is given as TP, and the true negative rate as TN.

**Figure 5:**
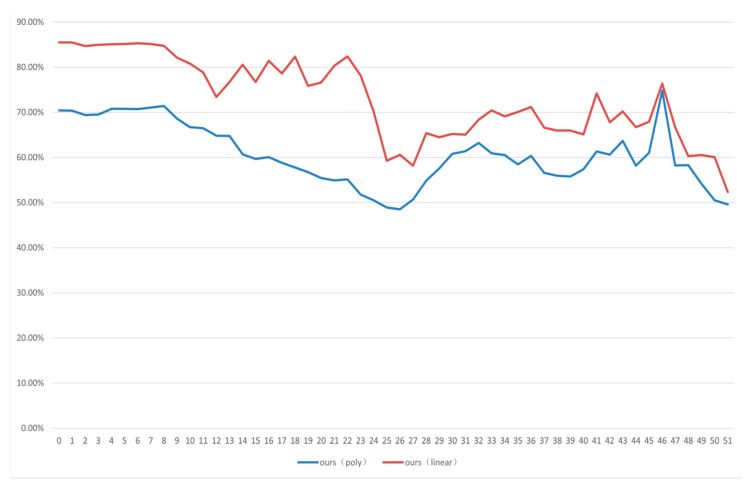
Security experiment results of the different quantization parameters (QPs).

In this experiment, 118 video sequences were randomly selected for SVM training, and the remaining 60 video sequences were used for classification. Different SVM kernels were processed in this experiment. The experiment was executed 10 times to calculate the average accuracy rate. As shown is Figure 5, the security of our algorithm has great performance, especially when the QP is bigger than 10. In addition, the security of our algorithm can maintain stabilization during different quantization parameters.

Since the security of the steganography must be compared with a similar capability, the payload of Nie et al.’s algorithm and Bouchama et al.’s algorithm was set to 0.375 and 0.5, respectively, so the capability of all the algorithms can be kept close. As shown in Table 6, the security of our algorithm is much better than Nie et al.’s algorithm and Bouchama et al.’s algorithm, in both experiment of 0.5 Mbit/s and 1.0 Mbit/s.

Furthermore, Wang et al.’s algorithm was also compared with our algorithm. Although Wang et al.’s algorithm is based on the HEVC encoding standard, in the security experiment of Wang et al.’s algorithm, Zhao et al.’s algorithm was also chosen as the steganalysis method. Since the encoding standard is different, the experiment is processed with different QPs, which is the same with Wang et al.’s paper. As shown in Table 7, the experiment result show that the security of our algorithm is greatly improved when the QP is smaller than 27. The security of our algorithm is slightly worse when the QP is bigger than 32. In addition, the security of Wang et al.’s algorithm will have huge variation during different quantization parameters, while the security of our algorithm can maintain stabilization during different quantization parameters.

## 6. Conclusions

A secure video steganography based on intra-prediction modes is proposed in this paper. In order to improve the performance of our algorithm, a novel embedding strategy is proposed. Different from adaptive steganography, the modification of the current block is processed immediately before the intra-prediction of the next block, so the modification of the current block will not affect the following blocks. In addition, after the modification of the IPM, the residual values will be modified to keep the optimality of the modified IPM. Finally, in order to keep the balance between the capability and video quality, a cover selection rule is proposed in this paper, where only the qualified blocks are embedded with a secret message. The experimental results show that this algorithm has great security performance and the security of our algorithm can basically maintain stabilization during different quantization parameters.

In the future work, we will improve the modification to the residual matrix, so the video quality will be better. The embedding method also will be improved to enhance the coding capability and security. In addition, the proposed algorithm will be implemented in HEVC since the HEVC encoding standard has become more and more popular all over the world.

## Figures and Tables

**Table 1:** Experimental results of the adaptive steganography embedding strategy.

Video Sequence	The Ratio of Optimal IPM Changing
akiyo.yuv	15.57%
bridge-close.yuv	18.90%
bridge-far_cif.yuv	19.54%
bus_cif.yuv	15.64%
coastguard_cif.yuv	17.47%
container_cif.yuv	12.96%
flower_cif.yuv	10.66%
foreman_cif.yuv	20.44%
hall_cif.yuv	13.23%
highway_cif.yuv	19.14%

**Table 2:** Security experiment results of the different thresholds.

Threshold	AR(Ploy)	AR(Linear)
16	46.52%	59.09%
32	46.52%	59.25%
48	49.96%	68.06%
64	55.03%	80.96%
80	56.05%	83.32%
90	66.18%	85.48%

**Table 3:** Video quality experimental results.

PSNR (AVG)	T = 96	T = 80	T = 64	T = 48	T = 32	T = 16
Akiyo.yuv	43.72	43.88	43.96	44.12	44.19	44.21
Bridge-close.yuv	33.8	33.76	33.8	33.75	33.87	33.87
Bus.yuv	28.24	28.26	28.29	28.27	28.27	28.30

**Table 4:** Video quality experimental results of the IDR frame.

PSNR (IDR)	T = 96	T = 80	T = 64	T = 48	T = 32	T = 16
Akiyo.yuv	40.71	41.77	41.93	42.46	42.66	42.66
Bridge-close.yuv	37.61	38.41	38.75	39.01	39.20	39.20
Bus.yuv	39.73	39.29	38.49	38.68	38.75	38.74

**Table 5:** Video quality experimental results.

PSNR	Bit Rate	No-E	Bouchama’s	Nie’s	Ours
Coastguard.yuv	0.5 m	28.14	28.03	28.06	28.00
	1 m	31.54	31.51	31.50	30.89
Mobile.yuv	0.5 m	29.01	28.83	28.86	28.76
	1 m	29.70	29.56	29.65	29.59
Waterfall.yuv	0.5 m	34.77	34.60	34.66	34.49
	1 m	37.95	37.82	37.90	37.86

**Table 6:** Security experiment results.

Bit Rate	Bouchama’s (payload 0.375)	Bouchama’s (payload 0.5)	Nie’s (payload 0.375)	Nie’s (payload 0.5)	Ours
0.5 Mbit/s	92.70%	93.80%	84.62%	86.70%	49.96%
1.0 Mbit/s	92.50%	95.60%	86.23%	88.20%	56.57%

**Table 7:** Security experiment results against Wang et al.’s algorithm.

QP	Ours	Wang’s
22	55.32%	90.85%
27	50.63%	80.37%
32	63.16%	59.93%
37	56.56%	56.03%
